# Supplementary material for: COPSOQ III in China: Preliminary Validation of an International Instrument to Measure Psychosocial Work Factors
Source: Healthcare (Basel). 2025 Apr 4;13(7):825. doi: 10.3390/healthcare13070825 (PMC11988307; doi:10.3390/healthcare13070825)
Supplement: Supplementary file 1 [file healthcare-13-00825-s001.zip › healthcare-3492010-supplementary.pdf]

File S1: Domains, Dimensions, and Items of the Chinese long-version of COPSOQ III in English/ Chinese.

Dimensions English/Chinese	Abbr.	Item name	Level	Question English/Chinese	Response options
Quantitative Demand/定量要求	QD	QD1*	MIDDLE	Is your workload unevenly distributed so it piles up? / 您的工作负荷被分配不均，因此其堆积成山吗？	1
		QD2	CORE	How often do you not have time to complete all your work tasks? / 由于时间不足，您无法完成您所有工作任务的频次是?	1
		QD3	CORE	Do you get behind with your work? / 您拖延您的工作了吗？	1
		QD4*	LONG	Do you have enough time for your work tasks? /您有足够的时间完成您的工作任务吗？	1R
Work Pace/工作节奏	WP	WP1	CORE	Do you have to work very fast? /您不得不很快地工作吗？	1
		WP2	CORE	Do you work at a high pace throughout the day? /您一整天都处于高节奏的工作吗？	2
		WP3	LONG	Is it necessary to keep working at a high pace? /保持高节奏的工作是必须的吗？	2
Cognitive Demand/认知要求	CD	CD1	LONG	Do you have to keep your eyes on lots of things while you work? /您工作时，不得不密切关注很多事情吗？	1
		CD2	LONG	Does your work require that you remember a lot of things?/您的工作要求您记住很多东西吗？	1
		CD3	LONG	Does your work demand that you are good at coming up with new ideas?/您的工作要求您擅长想出新思路吗？	1
		CD4	LONG	Does your work require you to make difficult decisions?/您的工作要求您做出艰难的决策吗？	1
Emotional Demand/情感要求	ED	ED1	MIDDLE	Does your work put you in emotionally disturbing situations?/您的工作使您处于情绪困扰的情境吗？	1

		EDX2	CORE	Do you have to deal with other people’s personal problems as part of your work?/作为您工作的一部分，您不得不处理他人的个人问题吗？	1
		ED3	CORE	Is your work emotionally demanding?/您的工作是情感要求型的吗？	2
Demands for Hiding Emotions/ 隐 藏 情绪的要求	HE	HE1	LONG	Are you required to treat everyone equally, even if you do not feel like it?/ 您的工作要求您平等对待每个人，即使您不喜欢这样吗?	1
		HE2	MIDDLE	Does your work require that you hide your feelings?/您的工作要求您隐藏您的感受吗？	2
		HE3	MIDDLE	Are you required to be kind and open towards everyone – regardless of how they behave towards/您的工作要求您对每个人都保持友善和开放的态度，无论他们如何待人吗?	2
		HE4*	MIDDLE	Does your work require that you do not state your opinion? /您的工作要求您不陈述您的意见吗？	1
Influence at Work/ 工作中的影响	IN	INX1	CORE	Do you have a large degree of influence on the decisions concerning your work?/您对涉及您工作的决策有很大程度的影响力吗？	1
		IN2*	LONG	Do you have a say in choosing who you work with?/您有选择与谁工作的发言权吗？	1
		IN3*	MIDDLE	Can you influence the amount of work assigned to you?/您能够影响分配给您的工作量吗？	1
		IN4*	MIDDLE	Do you have any influence on what you do at work?/您对您在工作中做什么有任何影响吗？	1
		IN5*	LONG	Can you influence how quickly you work? /您能够影响您工作（进度）多快吗？	1
		IN6*	MIDDLE	Do you have any influence on HOW you do your work?/在您的工作场所，您被清楚地提前告知涉及诸如未来的重要决策、变化或计划吗？	1

Possibilities for Development/发展的可能性	PD	PD2	CORE	Do you have the possibility of learning new things through your work?/通过工作，您有学习新生事物的可能性吗?	2
		PD3	CORE	Can you use your skills or expertise in your work?/您能够在您的工作中运用您的技能或专长吗？	2
		PD4*	MIDDLE	Does your work give you the opportunity to develop your skills?/您的工作给了您发展技能的机会吗？	2
Variation of Work/工作中的变化	VA	VA1	LONG	Is your work varied?/您的工作是变化的吗?	1
		VA2*	LONG	Do you have to do the same thing over and over again?/您不得不一遍又一遍做同样的事情吗？	1R
Control over Working time/对工作时间的掌控度	CT	CT1*	MIDDLE	Can you decide when to take a break?/您能够决定何时工间休息吗？	1
		CT2*	MIDDLE	Can you take holidays more or less when you wish?/您能根据自己的意愿或多或少地休假吗?	1
		CT3*	MIDDLE	Can you leave your work to have a chat with a colleague?/您能放下您的工作去和同事聊聊天吗?	1
		CT4*	MIDDLE	If you have some private business is it possible for you to leave your place of work for half an hour without special permission?/如果您有一些私事，您有可能离开您的工作场所半小时而无需专门许可吗？	1
		CT5*	LONG	Do you have to do overtime?/您不得不加班吗?	1R
Meaning of Work/工作的意义	MW	MW1	CORE	Is your work meaningful?/您的工作是有意义的吗?	2
		MW2*	MIDDLE	Do you feel that the work you do is important? /您感觉您所做的工作是重要的吗?	2
Predictability/可预测性	PR	PR1	CORE	At your place of work, are you informed well in advance concerning for example important decisions, changes or plans for the future?/在您的工作	2

				场所，您被良好地提前告知诸如关切未来的重要决策、变革或规划吗？	
		PR2	CORE	Do you receive all the information you need in order to do your work well?/为做好您的工作，您收到了所有您所需要的信息吗?	2
Recognition/认可	RE	RE1	CORE	Is your work recognized and appreciated by the management?/您工作场所的管理层尊重您吗？	2
		RE2	LONG	Does the management at your workplace respect you?/您的工作被管理层认可和赞赏吗?	2
		RE3	LONG	Are you treated fairly at your workplace?/在您的工作场所您是被公平对待的吗？	2
Role Clarity/角色明确度	CL	CL1*	CORE	Does your work have clear objectives?/您的工作有明确的目标吗？	2
		CL2	MIDDLE	Do you know exactly which areas are your responsibility?/您清楚地知道哪些领域是您负责的吗?	2
		CL3	MIDDLE	Do you know exactly what is expected of you at work? /您是否准确地知道（单位）对您工作的期望是什么吗？	2
Role Conflicts/角色冲突	CO	CO2	CORE	Are contradictory demands placed on you at work? /您有时不得不做一些本应已经以不同方式被完成的事情吗?	2
		CO3	CORE	Do you sometimes have to do things which ought to have been done in a different way? /在工作中，您被置于相互矛盾的要求之中吗？	2
Illegitimate Tasks/不合理的任务	IT	IT1*	MIDDLE	Do you sometimes have to do things which seem to be unnecessary?/您有时不得不做一些看似不必要的事情吗?	2
	QL	QL_T		To what extent would you say that your immediate superior…/您在多大程度上愿意说您的直接监管者……	

Quality of Leadership/领导力的质量		QLXI*	MIDDLE	-makes sure that the members of staff have good development opportunities?./确保其成员们有良好的发展机会？	2†
		QL2	LONG	-gives high priority to job satisfaction? /对工作满意度给予高度优先？	2†
		QL3	CORE	-is good at work planning? /擅长制定工作规划？	2†
		QL4	CORE	-is good at solving conflicts?/擅长解决冲突？	2†
Social Support from Supervisor/来自监管者的社会支持	SS	SSX1	MIDDLE	How often is your immediate superior willing to listen to your problems at work, if needed?/如果需要，您的直接领导多久愿意倾听您在工作中的问题?	1†
		SSX2	CORE	How often do you get help and support from your immediate superior, if needed?/如果需要，您多久从您的直接领导那里得到帮助和支持?	1†
		SSX3*	LONG	How often do you get help and support from your immediate superior, if needed?/您的直接领导会多久与您谈论您工作表现的好坏？	1†
Social Support from Colleagues/来自同事的社会支持	SC	SCX1	CORE	How often do you get help and support from your colleagues, if needed?/如果需要，您多久从您的同事那里得到帮助和支持?	1†
		SCX2	MIDDLE	How often are your colleagues willing to listen to your problems at work, if needed?/如果需要，您的同事多久愿意倾听您在工作中遇到的问题?	1†
		SC3*	LONG	How often do your colleagues talk with you about how well you carry out your work?/您的同事会多久与您讨论您工作表现的好坏？	1†
Sense of Community at Work 工作中/的社区意识	SW	SW1	CORE	Is there a good atmosphere between you and your colleagues?/您和您的同事之间有良好的氛围吗？	1†
		SW2*	LONG	Is there good co-operation between the colleagues at work?/在工作中，同事之间有良好的合作吗?	1†
		SW3*	MIDDLE	Do you feel part of a community at your place of work? /在您工作的地方，您感到您是社区的一部分吗?	1†

Commitment to the Workplace/对所在工作场所的承诺	CW	CW1	LONG	Do you enjoy telling others about your place of work?/您喜欢向别人介绍您工作的地方吗？	2
		CW2*	LONG	Do you feel that your place of work is of great importance to you?/您觉得您的工作场所对您来说非常重要吗？	2
		CWX3*	LONG	Would you recommend other people to apply for a position at your workplace?/您愿意推荐其他人在您的工作场所申请一个职位吗？	2
		CW4*	LONG	How often do you consider looking for work elsewhere?/您多频繁考虑要在其他地方找工作？	1R
		CW5*	LONG	Are you proud of being part of this organization?/您为作为这个组织的一员而骄傲吗？	2
Work Engagement/工作投入	WE	WE_T*		How often do you experience the following?/您多频繁经历一次如下（情况）：	
		WE1*	LONG	At my work, I feel bursting with energy/在我的工作中，我感到精力充沛。	3
		WE2*	LONG	I am enthusiastic about my job./您如下多久经历一次：我对我的岗位充满热情。	3
		WE3*	LONG	I am immersed in my work./您如下多久经历一次：我沉浸在我的工作中。	3
Job Insecurity/岗位无保障	JI	JI1	CORE	Are you worried about becoming unemployed? /您担心会失业吗?	2
		JI2*	LONG	Are you worried about new technology making you redundant?/您担心新技术会让您变得多余吗?	2
		JI3	CORE	Are you worried about it being difficult for you to find another job if you became unemployed?/您担心如果您失业，您会很难找到另一份工作吗?	2

Insecurity over Working Conditions/在工作条件上无保障	IW	IW1	CORE	Are you worried about being transferred to another job against your will?/您担心在违背您意愿的情况下被调离到另一个岗位吗？	2
		IW2*	LONG	Are you worried about your working tasks being changed against your will? /您担心在违背您的意愿的情况下变更您的工作任务吗？	2
		IW3*	MIDDLE	Are you worried about the timetable being changed (shift, weekdays, time to enter and leave ...) against your will？/您担心在违背您的意愿的情况下改变工作时间表（轮班、工作日、上下班时间...）吗？	2
		IW4*	MIDDLE	Are you worried about a decrease in your salary (reduction, variable pay being introduced ...)?/您担心您的工资会被减少（降薪、引入可变薪酬......）吗？	2
		IW5*	LONG	Are there good prospects in your job?/您的岗位有良好的前景吗？	2R
Quality of Work/工作质量	QW	QW1	LONG	To what extent do you find it possible to perform your work tasks at a satisfactory quality?/您发现在多大程度上可能以令人满意的质量完成您的工作任务？	2
		QW2	MIDDLE	Are you satisfied with the quality of the work performed at your workplace?/在您的工作场所，您对所完成工作的质量满意吗?	2
Job Satisfaction/岗位满意度	JS	JS_T		Regarding your work in general. How pleased are you with/对于工作总体而言，您有多满意	
		JS1	MIDDLE	-your work prospects? /您的工作前景？	6
		JS2	LONG	-the physical working conditions?/物理工作条件?	6
		JS3	LONG	-the way your abilities are used?/您的能力被使用的方式?	6
		JS4	CORE	-your job as a whole, everything taken into consideration?/将所有因素纳入考虑，您对整个工作满意吗?	6
		JS5*	MIDDLE	-your salary?/您的工资?	6

		WF_T		The next five questions concern the ways in which your work affects your private life:/以下五道问题涉及：哪些情况下您工作影响您的个人生活	
		WFX1*	LONG	Are there times when you need to be at work and at home at the same time?/有没有出现过您的工作和家里都同时需要您的情况?	1
		WF2	CORE	Do you feel that your work drains so much of your energy that it has a negative effect on your private life?/您感到您的工作空耗了您如此多的精力，以至于对您的私人生活产生了消极影响吗？	2
Work Life Conflict/工作—生活冲突	WF	WF3	CORE	Do you feel that your work takes so much of your time that it has a negative effect on your private life?/您感到您的工作占用了您如此多的时间，以至于对您的个人生活产生了消极影响吗？	2
		WF5*	LONG	The demands of my work interfere with my private and family life?/我的工作需求干扰了我的个人和家庭生活吗？	2
		WF6*	LONG	Due to work-related duties, I have to make changes to my plans for private and family activities./由于工作相关的职责，我不得不改变我的个人活动和家庭活动的计划。	2
(Intro Trust & Justice)				The next questions are not about your own job but about the workplace as a whole/接下来的问题不是关于你自己的工作，而是关于整个工作场所	
		TE1*	LONG	Do the employees withhold information from each other?/劳动者之间互相隐瞒信息吗？	2R
Horizontal Trust/横向信任	TE	TE2*	LONG	Do the employees withhold information from the management?/劳动者对管理层隐瞒信息吗？	2R
		TE3*	MIDDLE	Do the employees in general trust each other?/劳动者之间总体上是相互信任的吗？	2
Vertical Trust/纵向信任	TM	TM1*	CORE	Does the management trust the employees to do their work well? /管理层信任劳动者能做好他们的工作吗？	2

		TMX2*	CORE	Can the employees trust the information that comes from the management?/劳动者相信来自管理层的信息吗？	2
		TM3*	LONG	Does the management withhold important information from the employees?管理层对劳动者隐瞒了重要信息吗？	2R
		TM4*	MIDDLE	Are the employees able to express their views and feelings?劳动者能够表达他们的观点和感受吗？	2
Organizational Justice/组织公正	JU	JU1	CORE	Are conflicts resolved in a fair way?/冲突以公正的方式得以解决吗？	2
		JU2	LONG	Are employees appreciated when they have done a good job?/当劳动者工作做得很好时，他们是否受到赞赏？	2
		JU3	LONG	Are all suggestions from employees treated seriously by the management?/管理层是否认真对待劳动者的所有建议？	2
		JU4	CORE	Is the work distributed fairly?/工作的分配是否公平？	2
(Intro Negative Acts)/介绍消极行为				Conflicts and offensive behaviours/ 冲突和冒犯行为	
Gossip and Slander/流言和诽谤	GS	GS1	LONG	Have you been exposed to gossip and slander at your workplace during the last 12 months?/在过去的 12 个月期间，您曾在您的工作场所遭受过流言和诽谤吗？	4
		GS2	LONG	If yes, from whom? (You may tick off more than one)/如果是，来自谁？（您可以勾选多个选项）	5M
Conflicts and Quarrels/冲突和争吵	CQ	CQ1	LONG	Have you been involved in quarrels or conflicts at your workplace during the last 12 months?/在过去的 12 个月期间，您曾在您的工作场所被牵扯到冲突和争吵中吗？	4
Unpleasant Teasing/不愉快的取笑	UT	UT1	LONG	Have you been exposed to unpleasant teasing at your workplace during the last 12 months?/在过去 12 个月期间，您曾在您的工作场所遭受到不愉快的取笑吗？	4

		UT2	LONG	If yes, from whom? (You may tick off more than one)/如果是，来自谁？（您可以勾选多个选项）	5M
Cyber Bullying/网络欺凌	HSM	HSM1	LONG	Have you been exposed to work-related harassment on the social media (e.g. Facebook), by e-mail or text messages during the last 12 months?/在过去 12 个月期间，您曾在社交媒体（如微信/微博/QQ）上、通过电子邮件或短信遭受过与工作有关的骚扰吗？	4
		HSM2	LONG	If yes, from whom? (You may tick off more than one)/如果是，来自谁？（您可以勾选多个选项）	5M
Sexual Harassment/性骚扰	SH	SH1	LONG	Have you been exposed to undesired sexual attention at your workplace during the last 12 months?/在过去 12 个月期间，您曾在您的工作场所遭受过不想要的性关注吗？	4
		SH2	LONG	If yes, from whom? (You may tick off more than one)/如果是，来自谁？（您可以勾选多个选项）	5M
Threats of Violence/暴力威胁	TV	TV1	LONG	Have you been exposed to threats of violence at your workplace during the last 12 months?/在过去 12 个月期间，您曾在您的工作场所遭受过暴力的威胁吗？	4
		TV2	LONG	If yes, from whom? (You may tick off more than one)/如果是，来自谁？（您可以勾选多个选项）	5M
Physical Violence/身体暴力	PV	PV1	LONG	Have you been exposed to physical violence at your workplace during the last 12 months?/在过去 12 个月期间，您曾在您的工作场所遭受过身体暴力吗？	4
		PV2	LONG	If yes, from whom? (You may tick off more than one)/如果是，来自谁？（您可以勾选多个选项）	5M
Bullying/欺凌	BU	BU1	LONG	Bullying means that a person repeatedly is exposed to unpleasant or degrading treatment, and that the person finds it difficult to defend himself or herself against it. Have you been exposed to bullying at your workplace	4

				during the last 12months?/在过去 12 个月期间，您曾在您的工作场所遭受过欺凌吗？	
		BU3	LONG	If yes, from whom? (You may tick off more than one)如果是，来自谁？（您可以勾选多个选项）	5M
		BU2	LONG	How often do you feel unjustly criticized, bullied or shown up in front of others by your colleagues or your superior?/您多频繁感到被您同事或您领导不公正地批评、欺凌或在他人面前揭短？	1§
(Intro Health)/介绍健康				The following questions are about your own health and well-being. Please do not try to distinguish between symptoms that are caused by work and symptoms that are due to other causes. The task is to describe how you are in general.<br>The questions are about your health and well-being during the last four weeks:/以下问题是关于您的健康和幸福。请不要试图区分由工作引起的症状和由其他原因引起的症状。任务是描述您的总体情况。<br>这些问题是关于您在过去四周内的健康和福祉：	
Self Rated Health/自我健康评级	GH	GH1	LONG	In general, would you say your health is:/总体上，您认为您的健康是：	7
		GH2*	LONG	If you evaluate the best conceivable state of health at 10 points and the worst at 0 points: how many points do you then give your present state of health? /“如果您评估想象到的最好的健康状态是 10 分，最差的健康状态是 0 分：您给您现在的健康状况打多少分？”	8
Sleeping Troubles/睡眠问题	SL	SL_T	LONG	These questions are about how you have been during the last 4 weeks.	
		SL1*	LONG	How often have you slept badly and restlessly?/您多久有睡眠不好和睡眠不宁的情况？	9
		SL2	LONG	How often have you found it hard to go to sleep?您多久有难以入睡的情况？	9

		SL3	LONG	How often have you woken up too early and not been able to get back to sleep?/您多久有早醒而无法再入睡的情况?	9
		SL4	LONG	How often have you woken up several times and found it difficult to get back to sleep?/您多久有醒好几次并发现很难再入睡的情况？	9
Burnout/职业倦怠	BO	BO_T	LONG	These questions are about how you have been during the last 4 weeks./这些问题是关于您在过去 4 周的表现。	
		BO1	LONG	How often have you felt worn out./您多久感到疲惫不堪？	9
		BO2	LONG	How often have you been physically exhausted? /您多久感到身体上枯竭？	9
		BO3	LONG	How often have you been emotionally exhausted?/您多久感到情绪上枯竭？	9
		BO4	LONG	How often have you felt tired?/您多久感到劳累？	9
Stress/压力	ST	ST_T	LONG	These questions are about how you have been during the last 4 weeks./这些问题是关于您在过去 4 周的表现。	
		ST1	LONG	How often have you had problems relaxing?/您多久有难以放松的问题？	9
		ST2	LONG	How often have you been irritable?/您多久曾感到易怒？	9
		ST3	LONG	How often have you been tense?/您多久感到紧张？	9
Somatic Stress/躯体压力	SO	SO_T	LONG	These questions are about how you have been during the last 4 weeks./这些问题是关于您在过去 4 周的表现。	
		SO1*	LONG	How often have you had stomach ache?/您多久出现胃痛的症状？	9
		SO2*	LONG	How often have you had a headache?/您多久出现头痛的症状？	9
		SO3	LONG	How often have you had palpitations?/您多久出现心悸的症状？	9

		SO4	LONG	How often have you had tension in various muscles?/您多久出现各种肌肉紧张症状？	9
		CS_T	LONG	These questions are about how you have been during the last 4 weeks./这些问题是关于您在过去 4 周的表现。	
		CS1*	LONG	How often have you had problems concentrating?/您多久有注意力不集中的问题？	9
Cognitive Stress/认知压力	CS	CS2	LONG	How often have you found it difficult to think clearly?/您多久发现难以清晰思考？	9
		CS3	LONG	How often have you had difficulty in taking decisions?/您多久有难以做决定的情况？	9
		CS4	LONG	How often have you had difficulty with remembering?/您多久有记忆困难的情况？	9
		DS_T	LONG	These questions are about how you have been during the last 4 weeks./这些问题是关于您在过去 4 周的表现。	
		DS1	LONG	How often have you felt sad?/您多久感到悲伤？	9
Depressive Symptoms/抑郁症状	DS	DS2*	LONG	How often have you lacked self-confidence?/您多久缺乏自信？	9
		DS3*	LONG	How often have you had a bad conscience or felt guilty?/您多久感到良心不安或内疚？	9
		DS4*	LONG	How often have you lacked interest in everyday things?/您多久对日常事物缺乏兴趣？	9
Self-Efficacy/自我效能	SE	SE_T*		How well do these descriptions fit on you as a person?/以下描述符合您情况的程度有多大？	

	SE1*	LONG	I am always able to solve difficult problems, if I try hard enough./如果我足够努力的话，我总是有能力解决难题。这些描述对您有多好的契合度？	10
	SE2*	LONG	If people work against me, I find a way of achieving what I want./如果有人反对我，我总会找到办法实现我想要的。这些描述对您有多好的契合度？	10
	SE3*	LONG	It is easy for me to stick to my plans and reach my objectives./对我而言，坚持我的计划并达到我的目标是容易的。这些描述对您有多好的契合度？	10
	SE4*	LONG	I feel confident that I can handle unexpected events./我自信我能处理意外事件。这些描述对您有多好的契合度？	10
	SE5*	LONG	When I have a problem, I can usually find several ways of solving it./当我遇到问题时，我通常能找到几种办法加以解决。这些描述对您有多好的契合度？	10
	SE6	LONG	Regardless of what happens, I usually manage/无论发生什么事，我通常都能管理。这些描述对您有多好的契合度？	10

Note: * Deleted item after psychometric evaluation.

Note that CORE items are mandatory in all short, middle and long national versions of COPSOQ. Choice of items for national MIDDLE versions can deviate from the international version listed here.

*Response options explanation (and values for the scale - each scale is scored in the direction indicated by the scale name): 1: Always (100); Often (75); Sometimes (50); Seldom (25); Never/hardly ever (0)

1R: Always (0); Often (25); Sometimes (50); Seldom (75); Never/hardly ever (100) (Reversed scoring)

2: To a very large extent (100); To a large extent (75); Somewhat (50); To a small extent (25); To a very small extent (0)

2R: To a very large extent (0); To a large extent (25); Somewhat (50); To a small extent (25); To a very small extent (100) (Reversed scoring) 3: Never (0), Seldom (25), Sometimes (50), Often (75), Always (100)

4: Yes, daily; Yes, weekly; Yes, monthly; Yes, a few times; No

5M: Colleagues, Manager/superior, Subordinates, Clients/customers/patients (Multiple response options) 6: Very satisfied (100), Satisfied (75), Neither/Nor (50), Unsatisfied (25), Very unsatisfied (0)

7: Excellent (100), Very good (75), Good (50), Fair (25), Poor (0)

8: 0 (worst), 10, 20, 30, 40, 50, 60, 70, 80, 90, 100 (best)

9: All the time (100); A large part of the time (75); Part of the time (50); A small part of the time (25); Not at all (0) 10: Fits perfectly (100); Fits quite well (67); Fits a little bit (33); Does not fit (0)

†Including the response option, if deemed necessary: ‘I do not have a supervisor’ (coded as missing).

‡Including the response option, if deemed necessary: ‘I do not have colleagues’ (coded as missing).

§ Including the response option, if deemed necessary: ‘I do not have a superior / colleagues’ (coded as missing).

请注意，COPSOQ 的所有短篇、中篇和长篇版本中的核心项目都是强制性的。中级版本的项目选择可以与这里列出的国际版本不同。

*回答选项解释（以及量表的数值--每个量表的得分方向由量表名称指示）。

1: 总是（100）；经常（75）；有时（50）；很少（25）；从不/几乎没有 （0）

1R：总是（0）；经常（25）；有时（50）；很少（75）；从不/几乎没有（100）（负向计分）

2: 程度非常大（100）；程度很大（75）；有些（50）；程度很小（25）；程度非常小（0）

2R: 程度非常大（0）；程度很大（25）；有些（50）；程度很小（75）；程度非常小（100） 。

(反向计分）

3：从不（0），很少（25），有时（50），经常（75），总是（100）。

4: 是的，每天；是的，每周；是的，每月一次；是的，数次；不是

5M：同事、经理/上级、下属、客户/顾客/患者（您可以勾选多个选项）

6：非常满意（100），满意（75），中立（50），不满意 （25），非常不满意 （0）

7：优秀（100），非常好（75），好（50），一般（25），差（0）。

8: 0, 1, 2, 4, 5, 6 , 7, 8, 9, 10

9: 所有时间（100）；大部分时间（75）；部分时间（50）；一小部分时间 （25）；完全没有（0）

10: 完全符合（100）；相当符合（67）； 有一点符合（33）；不符合 （0）

如果认为有必要，包括回答选项："我没有领导"（编码为缺失）。

‡包括回答选项，如果认为有必要：'我没有同事'（编码为缺失）。

§ 如果认为有必要，包括回答选项：'我没有领导/同事'（编码为缺失）。

File S2: Differences of mean between the two groups with high and low scores

Dimensions	Abbr.	High scores group		Low scores group		t /CR	P
		Mean	SD	Mean	SD		
Quantitative Demands	QD1	64.65	20.51	20.00	14.51	30.01	<0.001
	QD2	61.75	18.94	16.67	12.53	33.52	<0.001
	QD3	58.68	21.22	13.07	13.85	30.39	<0.001
	QD4	51.23	21.36	16.23	17.73	21.28	<0.001
Work Pace	WP1	85.88	13.44	35.44	19.30	36.20	<0.001
	WP2	80.53	11.97	31.23	18.72	37.45	<0.001
	WP3	81.40	11.71	34.39	20.06	34.18	<0.001
Cognitive Demand	CD1	88.16	15.06	43.51	18.10	32.02	<0.001
	CD2	93.16	12.29	48.60	19.18	33.03	<0.001
	CD3	78.68	17.54	39.04	18.18	26.50	<0.001
	CD4	60.09	21.53	29.82	18.90	17.83	<0.001
Emotional Demand	ED1	65.18	19.23	21.84	16.35	28.99	<0.001
	EDX2	65.61	19.95	20.70	15.32	30.15	<0.001
	ED3	66.93	18.90	18.33	16.90	32.36	<0.001

Demands for Hiding Emotions	HE1	86.14	18.31	23.60	18.59	40.46	<0.001
	HE2	81.05	17.06	27.63	19.84	34.47	<0.001
	HE3	89.39	13.07	44.04	24.99	27.15	<0.001
	HE4	59.04	23.54	23.51	17.55	20.43	<0.001
Influence at Work	INX1	65.09	21.30	24.91	19.85	23.30	<0.001
	IN2	52.72	25.16	14.47	15.80	21.73	<0.001
	IN3	55.44	23.20	15.79	16.80	23.37	<0.001
	IN4	65.35	20.64	24.12	16.89	26.10	<0.001
	IN5	70.44	18.91	33.60	21.20	21.89	<0.001
	IN6	72.37	19.05	37.02	22.36	20.32	<0.001
Possibilities for Development	PD2	82.46	16.76	41.75	16.45	29.26	<0.001
	PD3	88.86	13.14	44.21	18.09	33.72	<0.001
	PD4	85.35	14.32	37.54	18.37	34.66	<0.001
Variation of Work	VA1	58.60	22.61	24.74	15.05	21.04	<0.001
	VA2	52.89	21.66	13.51	14.44	25.54	<0.001
Control over Working time	CT1	60.61	19.44	20.65	17.55	27.09	<0.001
	CT2	58.25	18.82	21.45	18.48	24.66	<0.001
	CT3	57.02	19.20	18.12	17.41	26.64	<0.001
	CT4	48.51	23.74	8.70	15.96	25.05	<0.001
	CT5	56.93	21.35	42.61	23.88	7.86	<0.001

Meaning of Work	MW1	97.98	6.82	45.88	14.33	55.44	<0.001
	MW2	98.95	5.03	42.19	16.06	56.93	<0.001
Predictability	PR1	84.04	13.08	29.39	20.51	37.92	<0.001
	PR2	83.42	13.41	37.92	37.92	32.19	<0.001
Recognition	RE1	86.67	14.61	45.18	20.47	27.86	<0.001
	RE2	85.44	13.38	36.67	18.82	35.66	<0.001
	RE3	87.28	12.87	38.07	19.85	35.12	<0.001
Role Clarity	CL1	91.23	12.84	46.14	14.78	38.88	<0.001
	CL2	97.46	8.40	44.39	16.89	47.50	<0.001
	CL3	90.79	12.44	43.51	14.89	41.13	<0.001
Role Conflicts	CO2	74.91	16.85	29.12	18.37	31.02	<0.001
	CO3	64.21	20.01	19.21	15.89	29.73	<0.001
Illegitimate Tasks	IT1	70.96	17.46	16.67	11.81	43.49	<0.001
Quality of Leadership	QLXI	82.63	14.42	36.93	21.14	30.15	<0.001
	QL2	84.82	13.27	39.47	20.63	31.21	<0.001
	QL3	82.46	14.51	37.28	20.73	30.14	<0.001
	QL4	81.40	14.71	37.54	20.41	29.43	<0.001
Social Support from Supervisor	SSX1	85.18	13.00	37.19	19.18	34.96	<0.001
	SSX2	85.35	12.51	39.30	19.33	33.76	<0.001
	SSX3	81.23	14.33	31.84	19.15	34.86	<0.001

Social Support from Colleagues	SCX1	86.75	13.02	43.07	15.35	36.64	<0.001
	SCX2	85.79	12.75	41.32	16.02	36.67	<0.001
	SC3	83.95	13.23	38.60	17.09	35.43	<0.001
Sense of Community at Work	SW1	95.61	9.98	45.88	14.48	47.76	<0.001
	SW2	95.96	9.21	46.49	15.30	46.76	<0.001
	SW3	85.00	18.67	41.46	18.19	28.17	<0.001
Commitment to the Workplace	CW1	81.40	18.43	36.32	17.96	29.58	<0.001
	CW2	89.82	12.83	45.35	17.50	34.60	<0.001
	CWX3	79.39	21.15	38.68	19.15	24.09	<0.001
	CW4	83.25	25.39	52.89	19.64	15.96	<0.001
	CW5	90.18	13.92	43.16	18.57	34.21	<0.001
Work Engagement	WE1	80.44	13.15	35.35	17.75	34.46	<0.001
	WE2	86.75	12.68	41.05	17.89	35.19	<0.001
	WE3	83.95	12.89	40.18	19.34	31.79	<0.001
Job Insecurity	JI1	71.14	21.35	17.28	16.44	33.74	<0.001
	JI2	64.39	24.36	17.63	17.25	26.44	<0.001
	JI3	73.16	22.18	21.32	18.99	29.98	<0.001
Insecurity over Working Conditions	IW1	77.02	20.08	24.39	19.28	31.93	<0.001
	IW2	76.58	19.28	22.98	16.73	35.45	<0.001
	IW3	73.16	22.28	22.63	18.79	29.27	<0.001

	IW4	86.05	16.93	36.23	24.22	28.46	<0.001
	IW5	50.44	7.50	33.86	7.50	7.50	<0.001
Quality of Work	QW1	83.07	12.61	45.00	14.81	33.04	<0.001
	QW2	85.26	12.67	44.74	14.94	34.93	<0.001
Job Satisfaction	JS1	86.40	12.99	45.44	16.69	32.70	<0.001
	JS2	83.07	13.94	41.14	17.50	31.64	<0.001
	JS3	83.95	13.06	44.56	14.42	34.18	<0.001
	JS4	84.39	13.00	47.19	13.47	33.54	<0.001
	JS5	77.37	15.59	39.30	17.79	27.17	<0.001
Work Life Conflict	WFX1	71.75	21.81	23.77	19.30	27.81	<0.001
	WF2	66.49	19.00	18.60	12.96	35.16	<0.001
	WF3	65.00	19.59	17.81	13.79	33.26	<0.001
	WF5	63.16	19.29	15.53	13.36	34.27	<0.001
	WF6	67.19	19.64	23.95	16.62	28.38	<0.001
Horizontal Trust	TE1	81.23	15.51	33.68	19.03	32.69	<0.001
	TE2	87.54	13.04	40.00	19.70	33.97	<0.001
	TE3	83.77	12.84	55.00	21.48	19.41	<0.001
Vertical Trust	TM1	85.35	14.77	44.74	17.39	30.05	<0.001
	TMX2	87.02	12.69	42.37	16.81	35.79	<0.001
	TM3	77.98	26.54	50.88	19.21	13.97	<0.001

	TM4	80.96	15.96	39.82	16.84	29.94	<0.001
Organizational Justice	JU1	84.65	14.04	42.72	16.84	32.29	<0.001
	JU2	86.84	12.85	41.84	16.56	36.24	<0.001
	JU3	85.70	12.74	40.09	18.42	34.38	<0.001
	JU4	82.54	14.54	41.67	17.13	30.71	<0.001
Self Rated Health	GH1	94.91	10.08	25.96	11.74	75.23	<0.001
Sleeping Troubles	SL1	70.00	16.49	17.72	13.00	38.80	<0.001
	SL2	65.79	17.82	15.88	12.42	40.57	<0.001
	SL3	65.88	18.29	12.37	12.70	37.19	<0.001
	SL4	62.37	19.75	10.79	12.58	37.19	<0.001
Burnout	BO1	65.00	16.81	16.75	12.50	38.88	<0.001
	BO2	61.23	16.55	11.67	12.67	40.14	<0.001
	BO3	59.91	17.31	10.79	13.43	37.85	<0.001
	BO4	64.82	15.89	18.77	12.35	38.63	<0.001
Stress	ST1	62.72	17.38	14.82	13.98	36.25	<0.001
	ST2	60.26	17.61	14.82	12.48	35.54	<0.001
	ST3	59.65	16.62	14.47	12.37	36.81	<0.001
Somatic Stress	SO1	53.25	19.24	3.16	8.83	39.94	<0.001
	SO2	55.88	17.38	6.05	11.13	40.76	<0.001
	SO3	53.68	17.67	3.33	8.77	43.10	<0.001

	SO4	54.47	18.28	2.37	7.33	44.66	<0.001
Cognitive Stress	CS1	57.11	17.55	11.67	12.84	35.27	<0.001
	CS2	56.14	16.97	7.63	11.72	39.71	<0.001
	CS3	54.12	16.60	9.74	13.25	35.28	<0.001
	CS4	61.49	17.85	8.77	13.51	39.76	<0.001
Depressive Symptoms	DS1	55.53	17.87	5.09	10.30	41.29	<0.001
	DS2	58.60	17.19	8.07	12.61	40.00	<0.001
	DS3	51.93	17.57	1.75	6.40	45.30	<0.001
	DS4	56.49	17.35	4.39	9.98	43.95	<0.001
Self-Efficacy	SE1	77.51	18.87	30.26	14.51	33.51	<0.001
	SE2	70.12	21.45	23.28	16.08	29.49	<0.001
	SE3	73.67	16.75	23.62	14.91	37.68	<0.001
	SE4	76.36	16.44	25.95	14.41	38.93	<0.001
	SE5	76.60	15.80	28.50	12.99	39.70	<0.001
	SE6	75.29	17.38	26.53	14.83	36.04	<0.001

File S3: The content validity results of the Chinese long-version of COPSOQ III.

Dimension	Abbreviation	Item name	Expert 1	Exper 2	Exper 3	Exper 4	Exper5	Exper 6	Exper 7	I-ICV
Quantitative Demands	QD	QD1	4	4	4	4	3	4	4	100.00
		QD2	4	4	4	4	4	3	4	100.00
		QD3	4	4	4	4	4	**2**	3	85.71
		QD4	4	4	4	4	4	3	4	100.00
Work Pace	WP	WP1	3	3	4	4	3	3	3	100.00
		WP2	3	3	4	4	4	3	4	100.00
		WP3	**2**	3	4	4	4	4	4	85.71
Cognitive Demands	CD	CD1	3	4	4	4	4	**2**	4	85.71
		CD2	3	4	4	4	4	3	3	100.00
		CD3	3	4	4	4	4	4	4	100.00
		CD4	3	4	4	4	4	4	4	100.00
Emotional Demands	ED	ED1	3	4	4	4	4	3	4	100.00
		EDX2	3	4	4	4	3	**2**	4	85.71
		ED3	3	4	4	4	4	3	4	100.00
Demands for Hiding Emotions	HE	HE1	**2**	3	4	4	4	4	4	85.71
		HE2	3	3	4	4	4	4	4	100.00
		HE3	**2**	3	4	4	4	3	4	85.71
		HE4	3	3	4	4	4	**2**	3	85.71
Influence at Work	IN	INX1	3	**2**	4	4	4	4	4	85.71

		IN2	**2**	**2**	4	4	4	3	3	71.43
		IN3	**2**	**2**	4	4	4	3	4	71.43
		IN4	**1**	**2**	4	4	3	**2**	4	57.14
		IN5	**2**	**2**	4	3	3	**2**	3	57.14
		IN6	**1**	**2**	4	4	4	3	4	71.43
Possibilities for Development	PD	PD2	3	4	4	4	4	3	**2**	85.71
		PD3	3	4	4	4	4	4	4	100.00
		PD4	3	4	4	4	4	4	4	100.00
Variation of Work	VA	VA1	3	**2**	4	4	3	3	3	85.71
		VA2	3	**2**	4	3	4	**2**	4	85.71
Control over Working time	CT	CT1	3	4	4	4	4	**2**	3	85.71
		CT2	3	4	4	4	4	**2**	4	85.71
		CT3	3	4	4	3	4	**2**	2	85.71
		CT4	3	4	4	3	3	**2**	**2**	71.43
		CT5	3	4	4	3	4	4	3	100.00
Meaning of Work	MW	MW1	**2**	4	4	**2**	4	4	4	71.43
		MW2	**2**	4	4	**2**	4	4	4	71.43
Predictability	PR	PR1	3	**2**	4	4	3	4	3	85.71
		PR2	3	**2**	4	4	3	4	3	85.71
Recognition	RE	RE1	3	4	4	4	4	4	4	100.00
		RE2	3	4	4	4	4	4	4	100.00
		RE3	3	4	4	4	4	3	4	100.00
Role Clarity	CL	CL1	3	4	4	4	4	4	3	100.00
		CL2	3	4	4	4	4	4	4	100.00
		CL3	3	4	4	4	4	4	3	100.00
Role Conflicts	CO	CO2	**2**	4	4	**2**	3	4	3	71.43

		CO3	3	4	4	4	4	3	3	100.00
Illegitimate Tasks	IT	IT1	3	4	4	3	4	3	3	100.00
Quality of Leadership	QL	QLXI	3	**2**	4	4	4	4	**2**	71.43
		QL2	3	**2**	4	4	4	3	3	85.71
		QL3	3	**2**	4	4	4	4	3	85.71
		QL4	3	**2**	4	4	4	4	3	85.71
Social Support from Supervisor	SS	SSX1	3	4	4	4	4	3	4	100.00
		SSX2	3	4	4	4	4	3	4	100.00
		SSX3	3	4	4	4	4	2	4	100.00
Social Support from Colleagues	SC	SCX1	3	4	4	3	4	3	4	100.00
		SCX2	3	4	4	3	4	4	2	100.00
		SC3	**2**	4	4	**2**	4	**2**	**2**	42.86
Sense of Community at Work	SW	SW1	3	4	4	4	4	3	4	100.00
		SW2	3	4	4	4	4	4	3	100.00
		SW3	**2**	4	4	**2**	4	**2**	**1**	42.86
Commitment to the Workplace	CW	CW1	3	4	4	4	4	4	4	100.00
		CW2	3	4	4	4	4	4	4	100.00
		CWX3	**2**	4	4	4	4	3	4	85.71
		CW4	**2**	4	4	4	4	3	4	85.71
		CW5	3	4	4	4	2	3	4	100.00
Work Engagement	WE	WE1	3	3	4	4	**2**	4	4	85.71
		WE2	3	3	4	4	2	3	4	100.00
		WE3	3	3	4	4	4	4	4	100.00
Job Insecurity	JI	JI1	3	4	4	4	4	4	4	100.00
		JI2	3	4	4	4	4	3	3	100.00
		JI3	3	4	4	4	**2**	3	3	85.71

Insecurity over Working Conditions	IW	IW1	3	4	4	4	4	4	4	100.00
		IW2	3	4	4	4	4	3	4	100.00
		IW3	3	4	4	4	3	3	4	100.00
		IW4	3	4	4	4	4	4	4	100.00
		IW5	3	4	4	4	4	3	3	100.00
Quality of Work	QW	QW1	3	4	4	4	4	4	**2**	85.71
		QW2	3	4	4	4	4	**2**	4	85.71
Job Satisfaction	JS	JS1	3	4	4	4	4	**2**	4	85.71
		JS2	4	4	4	4	4	3	4	100.00
		JS3	3	4	4	4	4	4	4	100.00
		JS4	3	4	4	3	4	4	4	100.00
		JS5	**2**	4	4	**2**	4	4	4	71.43
Work Life Conflict	WF	WFX1	**2**	4	4	3	4	**2**	**2**	57.14
		WF2	3	4	4	4	4	4	4	100.00
		WF3	3	4	4	4	4	3	4	100.00
		WF5	3	4	4	4	4	**2**	4	85.71
		WF6	3	4	4	4	4	**2**	4	85.71
Horizontal Trust	TE	TE1	**2**	4	4	3	4	3	3	85.71
		TE2	3	4	4	4	4	3	**2**	85.71
		TE3	3	4	4	4	4	4	4	100.00
Vertical Trust	TM	TM1	3	4	4	4	4	4	4	100.00
		TMX2	**2**	4	4	4	4	3	4	85.71
		TM3	**2**	4	4	4	4	3	4	85.71
		TM4	3	4	4	4	4	4	4	100.00
Organizational Justice	JU	JU1	3	4	4	4	4	3	4	100.00
		JU2	3	4	4	4	4	3	3	100.00

		JU3	3	4	4	4	4	3	4	100.00
		JU4	3	4	4	4	4	3	4	100.00
Gossip and Slander	GS	GS1	3	4	4	4	4	4	4	100.00
		GS2	3	4	4	4	4	4	4	100.00
Conflicts and Quarrels	CQ	CQ1	4	4	4	4	4	4	4	100.00
		CQ2	4	4	4	4	4	4	4	100.00
Unpleasant Teasing	UT	UT1	4	4	4	4	4	3	4	100.00
		UT2	4	4	4	4	4	3	4	100.00
Cyber Bullying	HSM	HSM1	4	4	4	4	4	4	4	100.00
		HSM2	4	4	4	4	4	4	4	100.00
Sexual Harassment	SH	SH1	4	4	4	4	4	4	4	100.00
		SH2	4	4	4	4	4	4	4	100.00
Threats of Violence	TV	TV1	4	4	4	4	4	4	4	100.00
		TV2	4	4	4	4	4	4	4	100.00
Physical Violence	PV	PV1	4	4	4	4	4	4	4	100.00
		PV2	4	4	4	4	4	4	4	100.00
		BU1	4	4	4	4	4	3	4	100.00
Bullying	BU	BU2	4	4	4	4	4	3	4	100.00
		BU3	4	4	4	4	4	3	4	100.00
Self Rated Health	GH	GH1	3	4	4	4	4	4	4	100.00
		GH2	3	4	4	4	4	4	**2**	85.71
		SL1	4	4	4	4	4	3	3	100.00
Sleeping Troubles	SL	SL2	4	4	4	4	4	**2**	3	85.71
		SL3	3	4	4	4	4	3	3	100.00
		SL4	3	4	4	4	4	3	3	100.00
Burnout	BO	BO1	3	4	4	4	3	3	3	100.00

		BO2	3	4	4	4	4	3	3	100.00
		BO3	3	4	4	4	4	3	3	100.00
		BO4	3	4	4	4	4	**2**	3	85.71
Stress	ST	ST1	3	4	4	4	4	3	3	100.00
		ST2	3	4	4	4	4	4	3	100.00
		ST3	3	4	4	4	4	3	3	100.00
Somatic Stress	SO	SO1	3	4	4	4	4	2	3	100.00
		SO2	3	4	4	4	4	3	4	100.00
		SO3	3	4	4	4	4	3	4	100.00
		SO4	3	4	4	4	4	3	3	100.00
Cognitive Stress	CS	CS1	3	4	4	4	4	**2**	**2**	71.43
		CS2	3	4	4	4	4	3	3	100.00
		CS3	3	4	4	4	4	3	3	100.00
		CS4	4	4	4	4	4	4	**2**	85.71
Depressive Symptoms	DS	DS1	4	4	4	4	3	4	4	100.00
		DS2	4	4	4	4	**2**	4	**2**	71.43
		DS3	3	4	4	4	4	4	3	100.00
		DS4	3	4	4	4	4	4	4	100.00
Self-Efficacy	SE	SE1	3	3	4	4	4	3	4	100.00
		SE2	3	3	4	4	4	**2**	4	85.71
		SE3	3	3	4	4	4	3	4	100.00
		SE4	3	3	4	4	4	3	4	100.00
		SE5	3	3	4	4	4	3	4	100.00
		SE6	3	3	4	4	4	**2**	4	85.71
S-CVC/Ave							92.52			

File S4: Reliability of the Chinese long-version of COPSOQ III.

Dimensions	Abbr.	Cronbach's α coefficient	CITC	CAID
Quantitative Demands	QD1	0.75	0.63	0.64
	QD2		0.69	0.61
	QD3		0.58	0.67
	QD4		0.31	0.81
Work Pace	WP1	0.81	0.63	0.77
	WP2		0.71	0.69
	WP3		0.65	0.76
Cognitive Demands	CD1	0.74	0.54	0.67
	CD2		0.58	0.65
	CD3		0.58	0.65
	CD4		0.42	0.74
Emotional Demands	ED1	0.75	0.58	0.65
	EDX2		0.59	0.64
	ED3		0.55	0.69
Demands for Hiding Emotions	HE1	0.75	0.56	0.70
	HE2		0.69	0.62
	HE3		0.55	0.70
	HE4		0.42	0.76
Influence at Work	INX1	0.78	0.54	0.74
	IN2		0.50	0.75
	IN3		0.54	0.74
	IN4		0.61	0.73
	IN5		0.52	0.75

	IN6		0.46	0.76
Possibilities for Development	PD2	0.79	0.58	0.78
	PD3		0.66	0.70
	PD4		0.68	0.67
Variation of Work	VA1	-	-	-
	VA2		-	-
Control over Working time	CT1	0.60	0.52	0.44
	CT2		0.49	0.47
	CT3		0.46	0.48
	CT4		0.39	0.52
	CT5		0.05	0.72
Meaning of Work	MW1	0.85	0.74	-
	MW2		0.74	-
Predictability	PR1	0.73	0.58	-
	PR2		0.58	-
Recognition	RE1	0.87	0.77	0.80
	RE2		0.75	0.82
	RE3		0.74	0.83
Role Clarity	CL1	0.85	0.70	0.81
	CL2		0.71	0.81
	CL3		0.76	0.76
Role Conflicts	CO2	0.68	0.51	-
	CO3		0.51	-
Illegitimate Tasks	IT1	-	-	-
Quality of Leadership	QLXI	0.84	0.72	0.78
	QL2		0.69	0.80

	QL3		0.68	0.80
	QL4		0.63	0.82
Social Support from Supervisor	SSX1	0.85	0.72	0.78
	SSX2		0.69	0.80
	SSX3		0.68	0.80
Social Support from Colleagues	SCX1	0.86	0.73	0.78
	SCX2		0.73	0.78
	SC3		0.70	0.81
Sense of Community at Work	SW1	0.79	0.73	0.82
	SW2		0.80	0.75
	SW3		0.70	0.85
Commitment to the Workplace	CW1	0.74	0.56	0.67
	CW2		0.68	0.63
	CWX3		0.50	0.70
	CW4		0.19	0.81
	CW5		0.65	0.64
Work Engagement	WE1	0.85	0.66	0.84
	WE2		0.78	0.72
	WE3		0.70	0.80
Job Insecurity	JI1	0.77	0.67	0.62
	JI2		0.59	0.71
	JI3		0.56	0.74
Insecurity over Working Conditions	IW1	0.78	0.72	0.67
	IW2		0.77	0.66
	IW3		0.68	0.69
	IW4		0.57	0.73

	IW5		0.09	0.87
Quality of Work	QW1	0.74	0.59	-
	QW2		0.59	-
	JS1		0.70	0.86
	JS2		0.70	0.85
Job Satisfaction	JS3	0.88	0.78	0.84
	JS4		0.76	0.84
	JS5		0.62	0.87
	WFX1		0.59	0.89
	WF2		0.79	0.84
Work Life Conflict	WF3	0.88	0.77	0.85
	WF5		0.79	0.84
	WF6		0.69	0.87
	TE1		0.18	0.78
Horizontal Trust	TE2	0.60	0.54	0.28
	TE3		0.55	0.27
	TM1		0.62	0.54
Vertical Trust	TMX2	0.69	0.68	0.50
	TM3		0.58	0.56
	TM4		0.13	0.84
	JU1		0.74	0.86
Organizational Justice	JU2	0.89	0.78	0.85
	JU3		0.79	0.84
	JU4		0.71	0.87
Sleeping Troubles	SL1	0.91	0.79	0.89
	SL2		0.82	0.89

	SL3		0.81	0.89
	SL4		0.80	0.89
Burnout	BO1	0.92	0.83	0.90
	BO2		0.86	0.89
	BO3		0.80	0.91
	BO4		0.81	0.91
Stress	ST1	0.87	0.73	0.84
	ST2		0.76	0.81
	ST3		0.77	0.80
Somatic Stress	SO1	0.88	0.71	0.86
	SO2		0.75	0.85
	SO3		0.78	0.84
	SO4		0.75	0.85
Cognitive Stress	CS1	0.91	0.79	0.89
	CS2		0.86	0.86
	CS3		0.79	0.89
	CS4		0.76	0.90
Depressive Symptoms	DS1	0.91	0.82	0.87
	DS2		0.81	0.88
	DS3		0.77	0.89
	DS4		0.77	0.89
Self-Efficacy	SE1	0.90	0.63	0.90
	SE2		0.65	0.89
	SE3		0.76	0.88
	SE4		0.79	0.87
	SE5		0.79	0.87

	SE6	0.75	0.88

Note: CITC: Corrected Item-Total Correlation; CAID: Cronbach's Alpha if Item Deleted.
